# Prognostic significance of Helix pomatia lectin and c-erbB-2 oncoprotein in human breast cancer.

**DOI:** 10.1038/bjc.1993.397

**Published:** 1993-09

**Authors:** M. Thomas, M. Noguchi, L. Fonseca, H. Kitagawa, K. Kinoshita, I. Miyazaki

**Affiliations:** Department of Surgery (II) Kanazawa University Hospital, Japan.

## Abstract

We investigated the prognostic significance of Helix pomatia lectin (HPA) staining on disease-free and overall survival in 120 primary breast carcinomas. HPA staining was present in 58 (48%) of these carcinomas. It was significantly associated with axillary lymph node metastases (P < 0.001) and c-erbB-2 expression (P < 0.01). A univariate study revealed that disease-free and overall survival were significantly correlated with clinical stage, tumour size, axillary lymph node metastases. HPA staining and c-erbB-2 expression. In a multivariate study, all previous prognostic indicators except HPA staining and c-erbB-2 expression were independent factors. However, stratifying the patients on the basis of HPA and c-erbB-2 status suggested that HPA +/c-erbB-2+ status was predictive of a higher incidence of axillary lymph node metastases (P = 0.000001) and a poorer overall (P < 0.0002) and a shorter disease-free (P < 0.000006) survival when compared with the other subgroups, although this combination did not provide any additional prognostic information for overall (P = 0.3544) or disease-free (P = 0.7152) survival by a multivariate analysis. For patients in whom axillary lymph node dissection has not been performed, therefore, HPA and c-erbB-2 status seems to be a powerful tool to discriminate subpopulations with a high recurrence risk and shorter survival who should undergo more aggressive therapy.


Br. J. Cancer (1993), 68, 621–626 © Macmillan Press Ltd., 1993

### Prognostic significance of *Helix pomatia* lectin and c-*erb*B-2 oncoprotein in human breast cancer

M. Thomas[1], M. Noguchi[1,2], L. Fonseca[1], H. Kitagawa[1], K. Kinoshita[1] & I. Miyazaki[1]

[1]*Department of Surgery (II) and* [2]*Operation Center, Kanazawa University Hospital, Takara-machi 13-1, Kanazawa, 920, Japan.*

**Summary** We investigated the prognostic significance of *Helix pomatia* lectin (HPA) staining on disease-free and overall survival in 120 primary breast carcinomas. HPA staining was present in 58 (48%) of these carcinomas. It was significantly associated with axillary lymph node metastases ($P<0.001$) and c-*erb*B-2 expression ($P<0.01$). A univariate study revealed that disease-free and overall survival were significantly correlated with clinical stage, tumour size, axillary lymph node metastases. HPA staining and c-*erb*B-2 expression. In a multivariate study, all previous prognostic indicators except HPA staining and c-*erb*B-2 expression were independent factors. However, stratifying the patients on the basis of HPA and c-*erb*B-2 status suggested that HPA+/c-*erb*B-2+ status was predictive of a higher incidence of axillary lymph node metastases ($P = 0.000001$) and a poorer overall ($P<0.0002$) and a shorter disease-free ($P<0.000006$) survival when compared with the other subgroups, although this combination did not provide any additional progostic information for overall ($P = 0.3544$) or disease-free ($P = 0.7152$) survival by a multivariate analysis. For patients in whom axillary lymph node dissection has not been performed, therefore, HPA and c-*erb*B-2 status seems to be a powerful tool to discriminate subpopulations with a high recurrence risk and shorter survival who should undergo more aggressive therapy.

The prognosis of patients with operable breast cancer is extremely variable. Recently, tremendous efforts have been made to assess the prognosis. The status of the axillary lymph nodes (AX) has been shown to be closely correlated with the prognosis (Haybittle *et al.*, 1982). Despite the proven prognostic value of nodal status, however, some centres have reduced the extent of AX dissection to avoid the related morbidity such as arm oedema. Therefore, there is a clear need to have accurate prognostic indices which do not involve axillary surgery.

In animal models, the ability of a tumour to metastasise may be determined through the selection of metastatic cell populations in which alterations in cell surface carbohydrates have been found to significantly alter the metastatic behaviour of the tumours (Altevogt *et al.*, 1983; Steck & Nicolson, 1983). Currently, such differences have been detected by means of lectins, which are sugar-binding proteins and glycoproteins of non-immune origin (Goldstein *et al.*, 1980; Leathem *et al.*, 1984; 1985) have described a *Helix pomatia* lectin (HPA) isolated from the albumin gland of the Roman snail, which recognises N-acetyl-galactosaminyl residues. It apparently binds to a population of breast cancer cells which have a higher frequency of metastases to the regional lymph nodes. Expression of HPA binding site in breast cancer tissue may reflect the ability of a tumour to invade and metastasise (Fiezy, 1981; Furmanski *et al.*, 1981).

The c-*erb*B-2 oncogene encodes a 185-kDa transmembrane receptor-like phosphoglycoprotein with tyrosine kinase activity (Coussens *et al.*, 1985; Akiyama *et al.*, 1986) that is closely related in structure, but is biologically distinct from, the epidermal growth factor receptor (EGFR, c-*erb*B-1) (Semba *et al.*, 1985; Yamamato *et al.*, 1986). It has been suggested that c-*erb*B-2 overexpression may play a significant role in the pathogenesis of breast disease (Berger *et al.*, 1988). The oncogene is amplified and overexpressed in about 15 to 30% of breast carcinomas (Slamon *et al.*, 1989; Paik *et al.*, 1990; Borg *et al.*, 1991). Several investigators have reported an association between c-*erb*B-2 activation and early disease recurrence or short survival, particularly in patients with AX-positive disease (Slamon *et al.*, 1989; Wright *et al.*, 1989; Borg *et al.*, 1991), whereas others have reported that c-*erb*B-2 has limited, if any, prognostic value (Van de Vijver *et al.*, 1988; Thor *et al.*, 1989).

Correspondence: M. Noguchi.
Received 6 January 1993; and in revised form 30 April 1993.

First in this study, we investigated the prognostic value of the HPA staining on disease-free and overall survival by univariate and multivariate analyses. Second, we attempted to assess whether HPA staining identified subsets of patients with a poorer prognosis when stratified on the basis of their c-*erb*B-2 status. Finally, we used multivariate analysis to ascertain whether the presence or absence of HPA staining contributed any additional prognostic information as a combined variable with c-*erb*B-2 status, to disease-free and overall survival models.

### Materials and methods

### *Patients and treatments*

One hundred and twenty paraffin blocks with tumour samples were available from patients with resectable breast cancer treated at the Department of Surgery II, Kanazawa University Hospital between 1978 and 1989. We selected only patients with histologically proven invasive breast carcinomas in whom AX dissection had been performed and had all resected material studied histologically. A variety of mastectomies was performed on all the patients. The resected breasts, pectoralis muscles, and the dissected axillary nodes were fixed in 10% formalin. Post-operatively, all patients underwent various adjuvant chemoendocrine therapies. They were followed until their death or through March, 1992.

The patients were classified according to the following clinico-pathological and biological variables: age ($\leqslant 35$ years in eight, 36–50 years in 61, $\geqslant 51$ years in 51), menopausal status (premenopausal in 61. postmenopausal in 59), clinical stage (stage 1 in 24, stage 2 in 69, stage 3 in 27), tumour size ($\leqslant 2.0$ cm in 44, 2.1–5,0 cm in 61, $\geqslant 5.1$ cm in 15), histologic type (invasive duct carcinoma in 112, special type of invasive carcinoma in eight according to The Histological Classification of Breast Cancer by the Japan Breast Cancer Society (1988) and the modified Histological Typing of the WHO (1982), degree of histological AX metastases (0 in 66, 1–3 in 26, $>3$ in 28), HPA binding (negative in 62, positive in 58) and c-*erb*B-2 expression (negative in 84, positive in 36).

### *HPA staining method*

The method used for preparation of the paraffin sections for HPA staining was similar to that of Fukutomi *et al.* (1989). Each section (5 μm-thick) was deparaffinised in xylene and subsequently rehydrated in ethanol and finally in water.

Endogenous peroxidase was blocked by immersion in methanol containing 1% hydrogen peroxide (15 min). After being washed in distilled water (5 min) and in phosphate buffered saline (PBS: pH 7.6) buffer (20 min), the sections were incubated with 10% normal porcine serum in PBS to reduce non-specific binding (30 min). They were then incubated overnight at room temperature with 5 $\mu g\ ml^{-1}$ diluted biotin-labelled HPA (Sigma, St Louis, MO, USA) in PBS buffer. Sections were washed three times for 5 min with PBS buffer between incubations. A final washing was followed by staining with a Vectastain ABC (Avidin-biotin-peroxidase complex) kit (Vector Laboratories Inc. Burlingame, CA, USA) (30 min). Peroxidase activity was visualised with 3.3'diaminobenzidine (DAB) and 1% hydrogen peroxide in PBS buffer (5 min). Sections were counterstained with 0.3% methyl green, dehydrated in ethanol cleared in xylene and mounted in malinol (Muto Pure Chemicals, Tokyo, Japan) medium. In negative control preparations the lectin was omitted. Most cases were clearly either intensely positive or completely negative. In a few cases we used the scoring described by Brooks *et al.* (1991). Finally, cases were classified as positively stained (HPA+) or negatively stained (HPA-).

*Immunohistochemical staining of c-erbB-2 oncoprotein*

The expression of c-*erb*B-2 oncoprotein was demonstrated in 5 μm sections, using a polyclonal antibody raised in rabbits against a synthetic peptide (21 N) representing residues 1243 to 1255 of the predicted oncoprotein sequence (Gullick *et al.*, 1987). The detailed procedure has been described elsewhere (Noguchi *et al.*, 1992). The tumours were scored by assessing the staining site (membrane and/or cytoplasm), the proportion of stained cells (0%, 1% to 49%, and 50% to 100%), and the intensity of staining (weak, +; strong, + +). If 50% or more of the tumour cells showed intense membrane staining, they were considered to be positive (c-*erb*B-2+). All other cells with less intense membrane staining or more focal staining were considered to be negative (c-*erb*B-2-).

*Statistical analysis*

The HPA binding and other variables were analysed using the Chi-squared test for significant association. Follow-up data were available for the disease-free survival and the overall survival. Disease-free and overall survival were calculated as the interval from the date of the operation to the recurrence or the death of the patient from breast cancer-related causes, respectively. For univariate analysis, the overall and disease-free survival were studied by the Kaplan-Meier method, and the log-rank test was used to analyse differences for significance. For multivariate analysis, Cox's life table regression model (proportional hazards general linear model) was used to examine several parameters simultaneously to test for their prognostic independence. Differences were considered significant when *P* was less than 0.05.

### Results

*Relationship of HPA staining with other prognostic variables*

Among the 120 cases studied, 62 (52%) had negative staining and 58 (48%) had positive staining. HPA staining was significantly associated with AX metastases ($P<0.001$) and c-*erb*B-2 expression ($P<0.01$), whereas it was not significantly associated with age, menopausal status, clinical stage, tumour size, and histologic type (Table I).

*Univariate and multivariate analyses on overall and disease-free survival*

When all the prognostic variables were examined individually with regard to overall and disease-free survival in the univariate model, there was a significantly increased risk of mortality and recurrence from breast cancer in the patients stratified by clinical stage ($P<0.01$), tumour size ($P<0.01$), AX metastases ($P<0.001$), HPA staining ($P<0.01$) and c-*erb*B-2 expression ($P<0.01$) (Table II). Life table analysis demonstrated an increased risk of earlier recurrence ($P<0.002$, Figure 1a) and shorter overall survival ($P<0.005$, Figure 1b) for the HPA+ group. The 10 year disease-free survival rate after surgery was 87% in the HPA- group and 60% in the HPA+ group. The survival curves for both groups displayed the same tendency (87% for the HPA- group and 63% for the HPA+ group). When all variables were considered simultaneously in the multivariate model to identify which variables conveyed unique prognostic information, the results indicated that all previous prognostic factors except HPA staining and c-*erb*B-2 expression were independent indicators for increased risk of mortality and recurrence (Table II).

*Relationship between axillary lymph node metastases and HPA and c-erbB-2 status*

We compared the presence of AX metastases with both HPA and c-*erb*B-2 status. Patients were categorised on the basis of HPA and c-*erb*B-2 status: (a) HPA- and c-*erb*B2-; (b) HPA- and c-*erb*B2+; (c) HPA+ and c-*erb*B2-; (d) HPA+ and c-*erb*B2+. Their relationship with AX metastases was highly significant ($P=0.000001$, Table III). The synchronous expression of HPA lectin and c-*erb*B-2 oncoprotein was more frequent in the group with a higher incidence of histological AX metastases (54%). Moreover, if we compared the patients grouped according to their HPA status, the overexpression of the c-*erb*B-2 oncoprotein

**Table I** Correlation of HPA staining with other prognostic factors in breast cancer (n = 120)

| *Variables* | *HPA positivity* | *P-value* | *Variables* | *HPA positivity* | *P-values* |
|---|---|---|---|---|---|
| Age | | | Tumor size | | |
| ≤ 35 yrs | 22% (2/9) | | ≤ 2.0 cm | 52% (23/44) | |
| 36-50 yrs | 46% (28/60) | NS | 2.1-5.0 cm | 49% (30/61) | NS |
| ≥ 51 yrs | 55% (28/51) | | ≥ 5.1 cm | 33% (5/15) | |
| Menopausal status | | | Degree of AX metastases | | |
| Pre | 40% (26/64) | NS | 0 | 30% (20/66) | |
| Post | 57% (32/56) | | 1-3 | 54% (14/26) | $P<0.001$ |
| Clinical stage | | | >3 | 86% (24/28) | |
| Stage 1 | 46% (11/24) | | c-erbB-2 expression | | |
| Stage 2 | 54% (37/69) | NS | c-erbB-2 (-) | 39% (33/84) | $P<0.01$ |
| Stage 3 | 37% (10/27) | | c-erbB-2 (+) | 69% (25/36) | |
| Histologic type | | | | | |
| Invasive ductal ca. | 50% (56/112) | | | | |
| Special type of invasive ca. | 25% (2/8) | NS | | | |

AX: Axillary lymph nodes.

**Table II** Univariate and Multivariate analysis of HPA staining and other prognostic factors on overall and disease-free survival ($n = 120$)

| | *Overall survival* | | *Disease-free survival* | |
|---|---|---|---|---|
| | *Univariate* | *Multivariate* | *Univariate* | *Multivariate* |
| Age | NS | NS | NS | NS |
| Menopausal | NS | NS | NS | NS |
| Clinical stage | <0.01 | <0.01 | <0.01 | <0.01 |
| Histologic type | NS | NS | NS | NS |
| Tumour size | <0.01 | <0.05 | <0.01 | <0.05 |
| AX metastases | <0.001 | <0.01 | <0.001 | <0.01 |
| HPA staining | <0.01 | NS | <0.01 | NS |
| c-erbB-2 expression | <0.01 | NS | <0.01 | NS |

AX: Axillary lymph nodes; NS: not significant.

**Table III** Axillary lymph node involvement related with HPA staining and c-erbB-2 expression

| *Ax*[a] | *HPA(−)c-erbB-2(−)* | *HPA(−)c-erbB-2(+)* | *HPA(+)c-erbB-2(−)* | *HPA(+)c-erbB-2(+)* | *Total* |
|---|---|---|---|---|---|
| 0 | 62% (41) | 8% (5) | 24% (16) | 6% (4) | 100% (66) |
| 1–3 | 31% (8) | 15% (4) | 31% (8) | 23% (6) | 100% (26) |
| >3 | 7% (2) | 7% (2) | 32% (9) | 54% (15) | 100% (28)[b] |
| Total | 42% (51) | 9% (11) | 28% (33) | 21% (25) | 100% (120) |

[a]Axillary lymph node metastases: [b]$P < 0.000001$.

**Figure 1** Actuarial survival curves for disease-free survival (**a**) and overall survival (**b**) for patients presenting with HPA positive or HPA negative staining primary breast carcinomas.

**Figure 2** Actuarial survival curves for disease-free survival (**a**) and overall survival (**b**) in the HPA/c-erbB-2 subgroups of patients with primary breast carcinomas.

identified a subset of patients with an increase incidence of AX metastases ($P<0.05$ for the HPA− group and $P<0.02$ for the HPA+ group, data not shown).

*Prognostic significance of HPA/c-*erbB-2 *status*

When patients were divided into the previous four groups (a to d), there was a significant trend for poorer overall survival ($P<0.0002$) and disease-free survival ($P<0.000006$) with HPA binding and c-*erb*B-2 overexpression (Figures 2a and b). The prognosis for Group (a) was significantly better than that for the other three groups (Figures 3a and b). The mean 10 year disease-free survival rate was 93% for Group (a) compared with 60, 80 and 39% for Groups (b), (c) and (d), respectively, whereas the mean 10 year overall survival rate was 93% for Group (a), 69% for Group (b), 79% for Group (c) and 46% for Group (d). Of the four survival and disease-free survival distributions, patients who were HPA+ and c-*erb*B2+ had a significantly worse disease outcome ($P<0.000006$, 10 year disease-free rate of 39%, Figure 2a) and shorter survival ($P<0.0002$, 10 year survival of 46%, Figure 2b) compared with the other groups; this suggests that HPA staining and c-*erb*B-2 expression are additive as prognostic indicators and may predict for a worse prognosis when both are positive. When the c-*erb*B-2 expression are additive as prognostic indicators and may predict for a worse prognosis when both are positive. When the c-*erb*B-2 oncoprotein is overexpressed, a subset of patients is defined among the HPA− group which had a shorter survival and earlier recurrence compared to the HPA+/c-*erb*B2 group (Figures 2a and b). No attempt was made to determine whether the combination of HPA staining and c-*erb*B-2 expression can identify group at higher recurrence risk in both AX-positive and AX-negative groups, because the number of cases was too small in this series.

To investigate whether the combination of HPA staining and c-*erb*B-2 oncoprotein expression gives any additional prognostic information for these patients, a multivariate analysis was conducted including all the prognostic factors selected in this study with the addition of the combination between HPA and c-*erb*B-2 status. However, this combination did not provide any additional prognostic information for overall ($P=0.3544$) or disease-free ($P=0.7152$) survival.

### Discussion

Our data and those from other laboratories (Leathem & Brooks, 1987; Fenlon *et al.*, 1987; Fukutomi *et al.*, 1989; Brooks & Leathem, 1991) indicate that HPA staining may be a valuable prognostic indicator in patients with primary breast cancer and may identify subsets of patients in defined prognostic groupings who have a worse prognosis. With regard to various clinicopathologic features, however, HPA staining has been associated with endocrine receptor status (Klein *et al.*, 1981; Fukutomi *et al.*, 1989), tumour stage (Leathem *et al.*, 1985) and high nuclear grade (Fukutomi *et al.*, 1989), implying an association between the HPA staining and enhanced malignancy of the tumour cells. The relationship between HPA binding and lymph node metastases is controversial, however it has been reported that HPA staining was significantly related to AX metastases (Leatherm *et al.*, 1984; Fenlon *et al.*, 1987), although other reports have found no significant relationship between them (Fukutomi *et al.*, 1989; Galea *et al.*, 1991). Moreover, using flow cytometry and fresh breast cancer tissues, Alam *et al.* (1990) found a correlation between HPA staining and nodal involvement ($P<0.001$). This was confirmed in this study by the finding that the majority of the HPA+ tumours were associated with the presence of AX metastases. By multivariate analysis, there appears to be a significant relationship between HPA staining and AX metastases since the prognostic significance of HPA staining was lost when nodal status was introduced into the model. This confounding effect between both prognostic factors has been found by Brooks and Leathem (1991) but not by Fukutomi *et al.* (1989) who found that HPA binding is a more informative prognostic factor for overall survival than lymph node involvement. Although the incidence of PHA staining (48%) in this study is similar to that of Fukutomi *et al.* (1989) but not in agreement with the 80% incidence reported by Brooks *et al.* (1991), the difference is less likely to be due to the type of staining method or source of lectin than to the overall sensitivity of the method. This may also have some general bearing on the significance of HPA staining.

On the other hand, the function of the c-*erb*B-2 protein in normal growth and differentiation of tumour cells remains unclear. Its 50% amino acid sequence homology to EGFR (Yamamoto *et al.*, 1986) suggests that the c-*erb*B-2 receptor may behave similarly to EGFR and be a potent growth stimulator. Several studies (Tandon *et al.*, 1989; Wright *et al.*, 1989; McCann *et al.*, 1991) have reported that c-*erb*B-2 may be a potential predictor for the course of the disease in breast carcinoma patients. Amplification and overexpression of this gene occurs more often in AX-positive patients (Thor *et al.*, 1989; Borg *et al.*, 1991) as well as in tumours characterised by high grade (Paik *et al.*, 1990; Paterson *et al.*, 1991), large size (Borg *et al.*, 1991) or absence of oestrogen and progesterone receptors (Thor *et al.*, 1989; Borg *et al.*, 1991; Lovekin *et al.*, 1991). In our previous study (Noguchi *et al.*, 1992), c-*erb*B-2 oncoprotein overexpression was significantly associated with clinical stage and AX metastases.

In the present study, both HPA staining and c-*erb*B-2 overexpression were found to be strongly associated with the presence of AX metastases. Moreover, we found that the c-*erb*B-2 status was predictive of a worse disease outcome and a higher recurrence rate in both the HPA+ and HPA− groups; however, of most interest is our finding that the HPA+/c-*erb*B2+ patients had the worst prognosis when compared with the other subgroups. Such a combined effect has been reported for c-*erb*B2+ and EGFR-positive patients (Wright *et al.*, 1989). Thus, among patients regarded as having a more favourable prognosis (HPA− group), c-*erb*B-2 staining identified those who might benefit from more aggressive therapy at an early stage. When HPA was assessed against c-*erb*B-2, a strong association has been found ($P<0.01$) contrary to Galea *et al.* (1991) who found no relation between HPA binding and all prognostic factors tested (including c-*erb*B-2 status) except NCRC-11 (anti-polymorphic epithelial mucin antibody). A possible explanation for discrepancy may lie in differences in the staining method and source of HPA; Galea *et al.* (1991) used a direct peroxidase conjugated *Helix pomatia* method. In the future, a study of the relationship between HPA staining and the S-phase fraction will be required to determine whether HPA staining is correlated with the proliferative ability of a tumour. Since tumours with high proliferative activity may have good responses to therapy (Bonnadonna *et al.*, 1986), HPA staining may assist in determining treatment strategies for particular groups of patients.

In conclusion, we have shown that HPA staining is a significant predictor for poorer disease-free and overall survival in primary breast carcinoma patients, although it was not significant by multivariate analysis. However, one should consider not only the status of HPA staining, but also the status of the c-*erb*B-2 oncoprotein to discriminate more precisely sub-populations with a high recurrence risk and short survival who might benefit from more aggressive therapy. This combination would provide a valuable prognostic information for breast cancer patients in whom axillary lymph node dissection has not been performed.

We would like to thank R. Yashiki and N. Takamura for their excellent technical assistance. M.T. is a recipient of a fellowship from the Japanese–Germany Center Berlin (Berlin, Germany).

### References

AKIYAMA, T., SUDO, C., OGAWARA, H., TOYOSHIMA, K. & YAMAMOTO, T. (1986). The product of the human c-*erb*B-2 gene: A 185-kilodalton glycoprotein with tyrosine kinase activity. *Science (Washington DC)*, **232**, 1644–1646.

ALAM, S.M., WHITFORD, P., CUSHLEY, W., GEORGE, W.D. & CAMPBELL, A. (1990). Flow cytometric analysis of cell surface carbohydrates in metastatic human breast cancer. *Br. J. Cancer*, **62**, 238–242.

ALTEVOGT, P., FOGEL, M., CHEINGSONG-POPOV, R., DENNIS, J., ROBINSON, P. & SCHIRRMACHER, V. (1983). Different patterns of lectin binding and cell surface sialylation detected on related high- and low-metastatic tumor lines. *Cancer Res.*, **43**, 5138–5144.

BERGER, M.S., LOCHER, G.W., SAURER, S., GULLICK, W.J., WATERFIELD, M.D., GRONER, B. & HYNES, N.E. (1988). Correlation of c-*erb*B-2 gene amplification and protein expression in human breast carcinoma with nodal status and nuclear grading. *Cancer Res.*, **48**, 1238–1243.

BONNADONNA, G., VALAGUSSA, P., TANCINI, G., ROSSI, A., BRAMBILLA, C., ZAMBETTI, M., BIGNAMI, P., DI FRONZO, G. & SILVESTRINI, R. (1986). Current status of Milan adjuvant chemotherapy trials for node-positive and node-negative breast cancer. *Natl Cancer Inst. Monogr.*, **1**, 45–49.

BORG, A., DALDETORP, B., FERNO, M., KILLANDER, D., OLSSON, H. & SIGURDSSON, H. (1991). ERBB2 amplification in breast cancer with high rate of proliferation. *Oncogene*, **6**, 137–143.

BROOKS, S.A. & LEATHEM, A.J. (1991). Prediction of lymph node involvement in breast cancer by detection of altered glycosylation in the primary tumour. *Lancet*, **338**, 71–74.

COUSSENS, L., YANG-FENG, T.L., LIAO, Y.C., MCGRATH, J., SEEBURG, P.H., LIBERMANN, T.A., SCHLESSINGER, J., FRANCKE, U., LEVISON, A. & ULLRICH, A. (1985). Tyrosine kinase receptor with extensive homology to EGF receptor shares chromosomal location with neu oncogene. *Science (Washington DC)*, **230**, 1132–1139.

FENLON, S., ELLIS, I.O., BELL, J., TODD, J.H., ELSON, D.W. & BLAMEY, R.W. (1987). *Helix pomatia* and *Ulex europeus* lectin binding in human breast carcinoma. *J. Pathol.*, **152**, 169–176.

FIEZI, T. (1981). Carbohydrate differentiation antigens. *Trends Bioch. Sci.*, **6**, 333–335.

FUKUTOMI, T., ITABASHI, M., TSUANE, S., YAMAMOTO, H., NANASAWA, T. & HIROTO, T. (1989). Prognostic contributions of Helix pomatia and carcinoembryonic antigen staining using histochemical techniques in breast carcinomas. *Jpn. J. Clin. Oncol.*, **19**, 127–134.

FURMANSKI, P., KIRKLAND, W.L., GARGALA, T. & RICH, M.A. (1981). The breast cancer prognostic study clinical associates, prognostic value of concanavlin A reactivity of primary breast cancer cells. *Cancer Res.*, **42**, 4087–4092.

GALEA, M.H., ELLIS, I.O., BELL, J., ELSTON, C.W. & BLAMEY, R.W. (1991). Prediction of lymph node involvement in breast cancer. *Lancet*, **328**, 392–393.

GOLDSTEIN, I.J., HUGHES, R.C., MONSIGNY, M., OSAWA, T. & SHARON, N. (1980). What should be called a lectin? *Nature*, **285**, 66.

GULLICK, W.J., BERGER, M.S., BENNETT, P.L.P., ROTHBARD, J.R. & WATERFIELD, M.D. (1987). Expression of the c-erbB-2 protein in normal and transformed cells. *Int. J. Cancer*, **40**, 246–254.

HAYBITTLE, J.L., BLAMEY, R.W., ELSTON, C.W. & others. (1982). A prognostic index in primary breast cancer. *Br. J. Cancer*, **45**, 361–366.

JAPAN BREAST CANCER SOCIETY (1988). *General rules for clinical and pathological record of breast cancer tumors.* ed. 9 Tokyo: Kinbara Co.

KLEIN, P.J., VIERBUCHEN, M., WURZ, H., SCHULZ, K.D. & NEWMAN, R.A. (1981). Secretion-associated lectin–binding sites as a parameter of hormone dependence in mammary carcinoma. *Br. J. Cancer*, **44**, 746–748.

LEATHEM, A., ATKINS, N. & EISEN, T. (1985). Breast cancer metastasis, survival and carbohydrate expression associated with lectin binding. *J. Pathol.*, **145**, 73A.

LEATHEM, A., DOKAL, I. & ATKINS, N. (1984). Carbohydrate expression in breast cancer as an early indicator of metastatic potential. *J. Pathol.*, **142**, A32.

LEATHEM, A.J. & BROOKS, S.A. (1987). Predictive value of lectin binding on breast-cancer recurrence and survival. *Lancet*, **1**, 1054–1056.

LOVEKIN, C., ELLIS, I.O., LOCKER, A., ROBERTSON, J.F.R., BELL, J., NICHOLSON, R., GULLICK, W.J., ELSTON, C.W. & BLAMEY, R.W. (1991). c-erbB-2 oncoprotein expression in primary and advanced breast cancer. *Br. J. Cancer*, **63**, 439–443.

MCCANN, A.H., DERVAN, P.A., O'REGAN, M., CODD, M.B., GULLICK, W.J., TOBIN, B.M.J. & CARNEY, D.N. (1991). Prognostic significance of c-erbB-2 and estrogen receptor status in human breast cancer. *Cancer Res.*, **51**, 3296–3303.

NOGUCHI, M., KOYASAKI, N., OHTA, N., KITAGAWA, H., EARASHI, M., THOMAS, M., MIYAZAKI, I, & MIZUKAMI, Y. (1992). C-erbB-2 oncoprotein overexpression versus internal mammary lymph node metastases as additional prognostic factors in patients with axillary lymph node-positive breast cancer. *Cancer*, **69**, 2953–2960.

PAIK, S., HAZAN, R., FISHER, E.R., SASS, R.E., FISHER, B., REDMOND, C., SCHLESSINGER, L., LIPPMAN, M.E. & KING, C.R. (1990). Pathologic findings from the national surgical adjuvant breast and bowel project: prognostic significance of c-erbB-2 protein over-expression. *J. Clin. Oncol.*, **8**, 103–112.

PATERSON, M.C., DIETRICH, K.D., DANYLUK, J., PATERSON, A.H.G., LEES, A.W., JAMIL, N., HANSON, J., JENKINS, H., KRAUSE, B.E., MCBLAIN, W.A., SLAMON, D.J. & FOURNEY, R.M. (1991). Correlation of c-erbB-2 amplification and risk of recurrent disease in node-negative breast cancer. *Cancer Res.*, **51**, 556–567.

SEMBA, K., KAMATA, N., TOYOSHIMA, K. & YAMAMOTO, T.A. (1985). v-erbB related protooncogene, c-erbB-2, is distinct from the c-erbB-1/epidermal growth factor-receptor gene and is amplified in a human salivary gland adenocarcinoma. *Proc. Natl Acad. Sci. USA*, **82**, 6497–6501.

SLAMON, D.J., GODOLPHIN, W., JONES, L.A., HOLT, J.A., WONG, S.G., KEITH, D.E., LEVIN, W.J., STUART, S.G., UDOVE, J., ULLRICH, A. & PRESS, M.F. (1989). Studies of the HER-2/neu proto-oncogene in human breast and ovarian cancer. *Science (Wash. DC)*, **244**, 707–712.

STECK, R.A. & NICOLSON, G.I. (1983). Cell surface glycoprotein of 13762 NF mammary adenocarcinoma clones of differing metastatic potentials. *Exp. Cell Res.*, **147**, 255–267.

TANDON, A.K., CLARK, G.M., CHAMNESS, G.C., ULLRICH, A. & MCGUIRE, W.I. (1989). HER-2/neu oncogene protein and prognosis in breast cancer. *J. Clin. Oncol.*, **7**, 1120–1128.

THE WORLD HEALTH ORGANIZATION (1982). The World Organization for histological typing of breast tumors – second edition. *Am. J. Clin. Pathol.*, **78**, 806–816.

THOR, A.D., SCHWARTZ, L.H., KOERNER, F.C., EDGERTON, S.M., SKATES, S.J., YIN, S., MCKENZIE, S.J., PANICALI, D.I., MARKS, P.J., FINGERT, H.J. & WOOD, W.C. (1989). Analysis of c-erbB-2 expression in breast carcinomas with clinical follow-up. *Cancer Res.*, **49**, 7147–7152.

VAN DE VIJVER, M.J., PETERSE, J.L., MOOI, W.J., WISMAN, P., LOMANS, J., DALESIO, O. & NUSSE, R. (1988). Neu-protein overexpression in breast cancer. Association with comedo-type ductal carcinoma *in situ* and limited prognostic value in stage II breast cancer. *N. Engl. J. Med.*, **319**, 1239–1245.

WRIGHT, C., ANGUS, B., NICHOLSON, S., SAINSBURY, J.R.C., CAIRNS, J., GULLICK, W.J., KELLY, P., HARRIS, A.L. & HORNE, C.H.W. (1989). Expression of c-erbB-2 oncoprotein: a prognostic indicator in human breast cancer. *Cancer Res.*, **49**, 2087–2090.

YAMAMOTO, T., IKAWA, S., AKIYAMA, T., SEMBA, K., NOMURA, N., MIYAJIMA, N., SAITO, T. & TOYOSHIMA, K. (1986). Similarity of protein encoded by the human c-erbB-2 gene to epidermal growth factor receptor. *Nature*, **319**, 230–234.


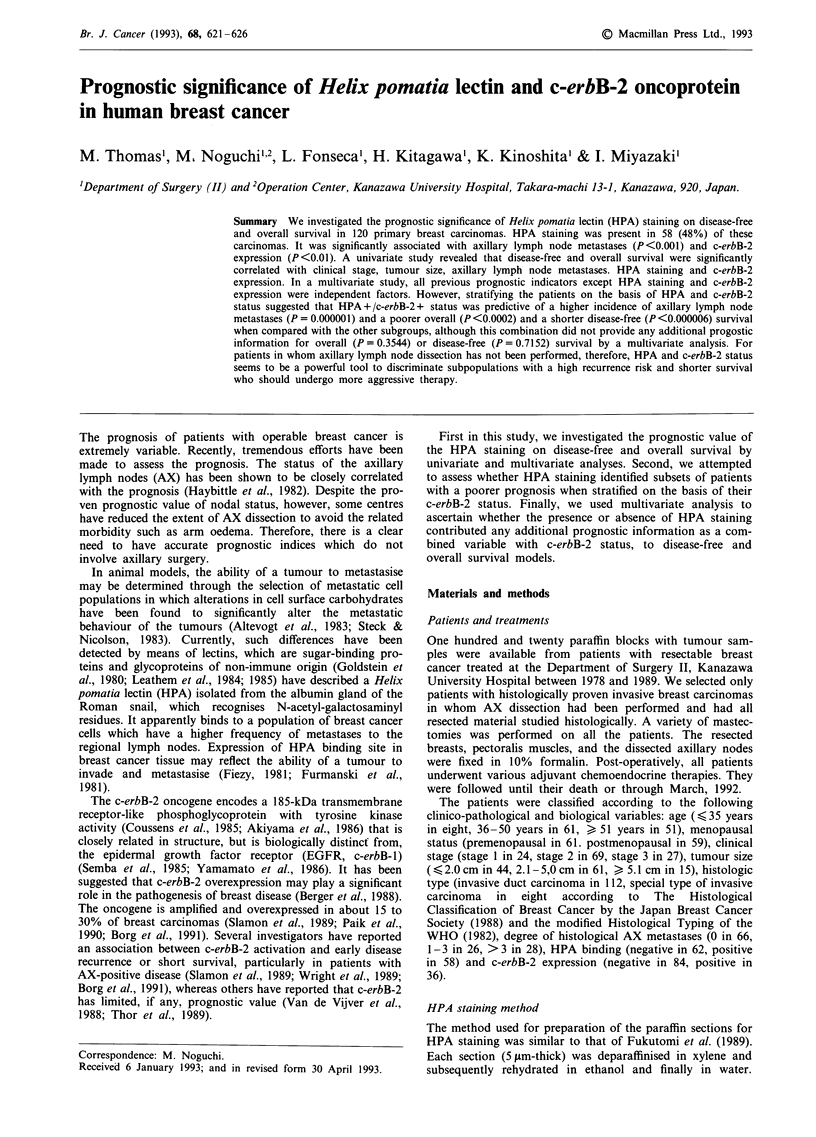

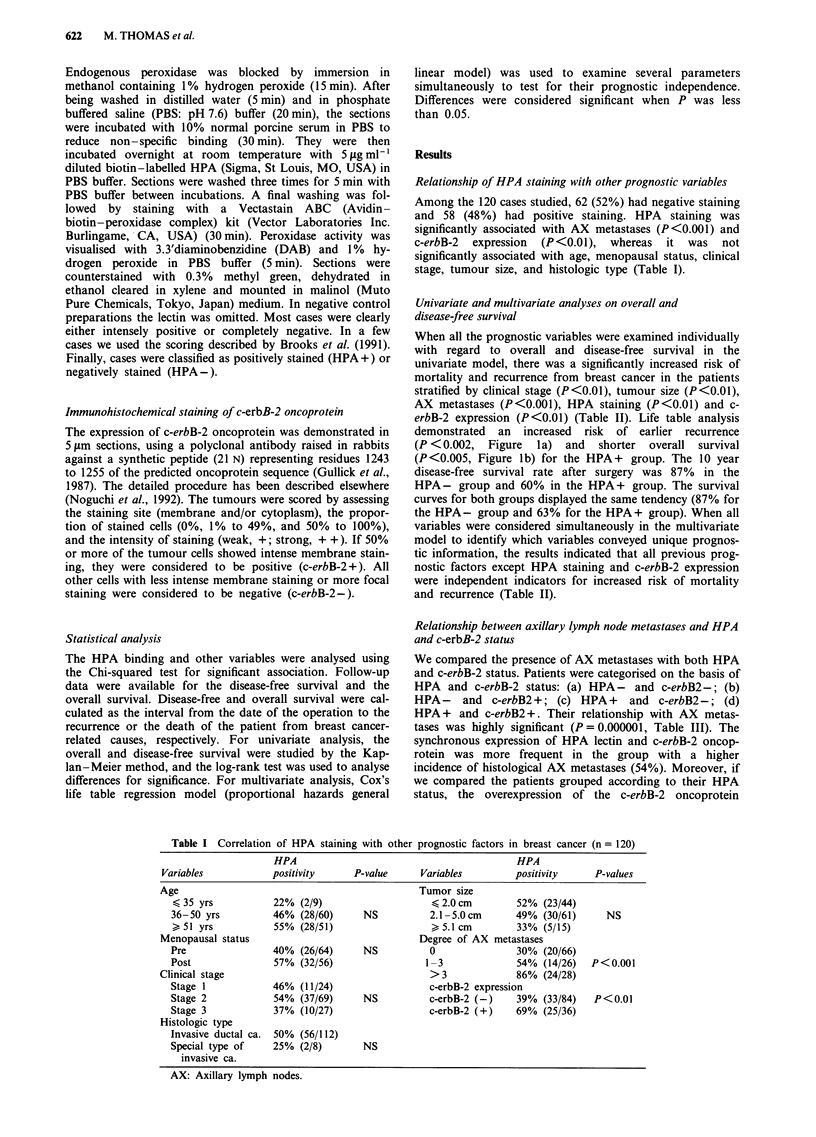

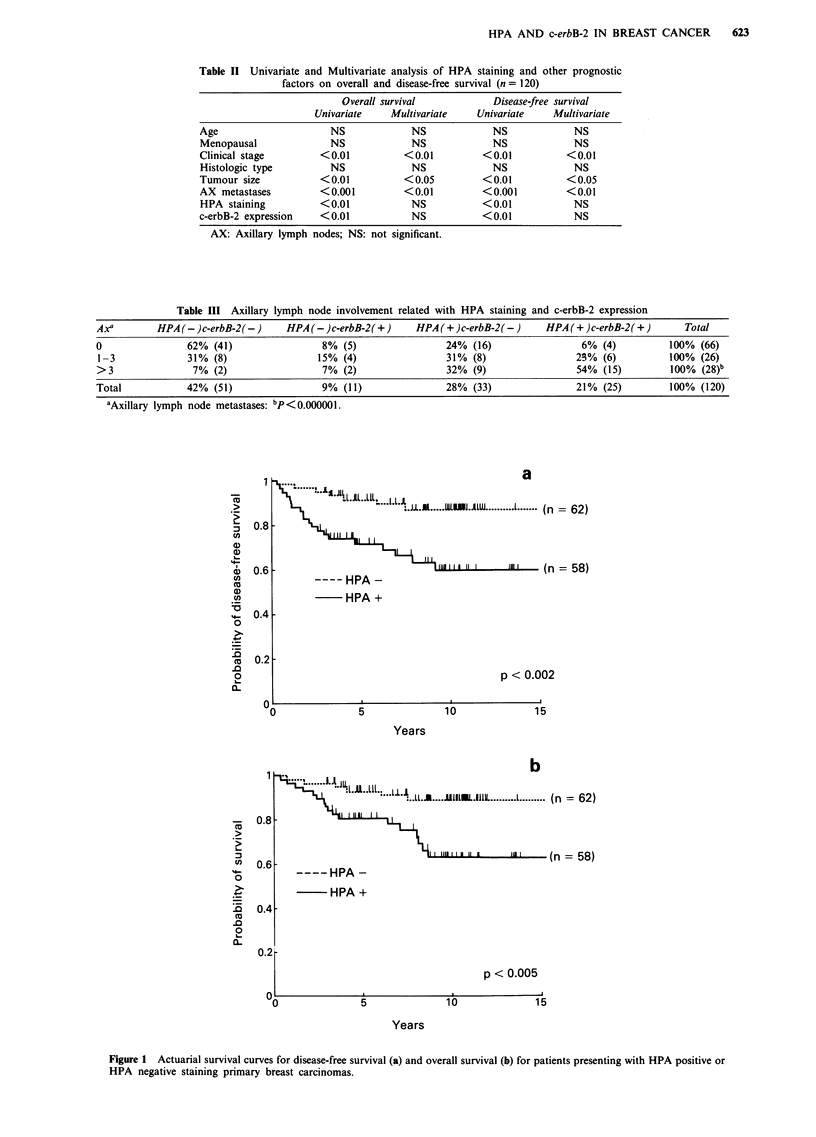

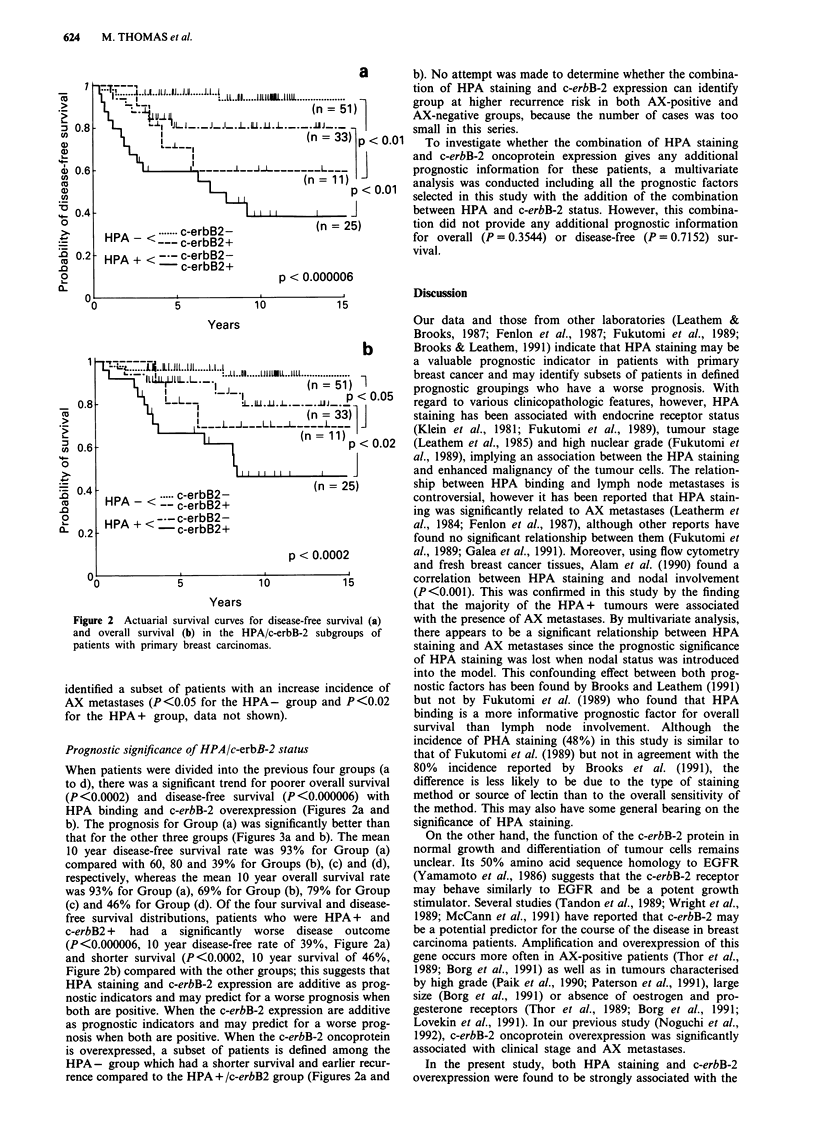

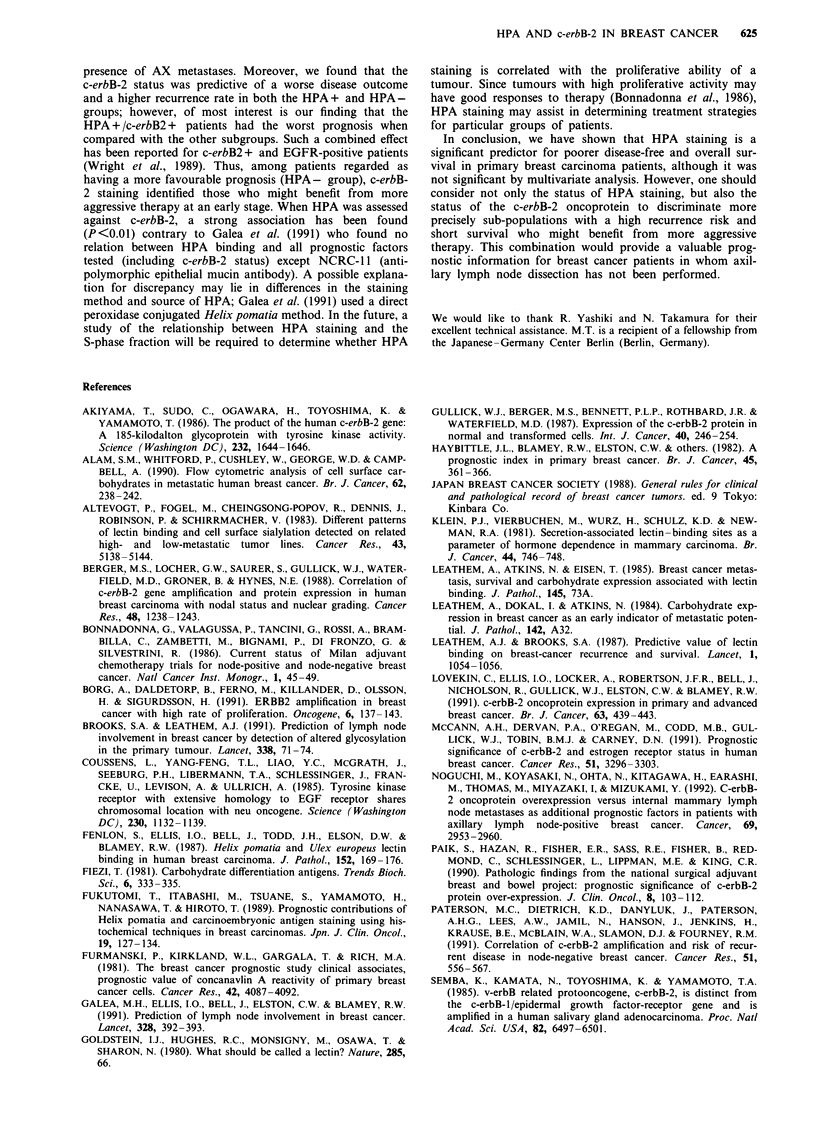

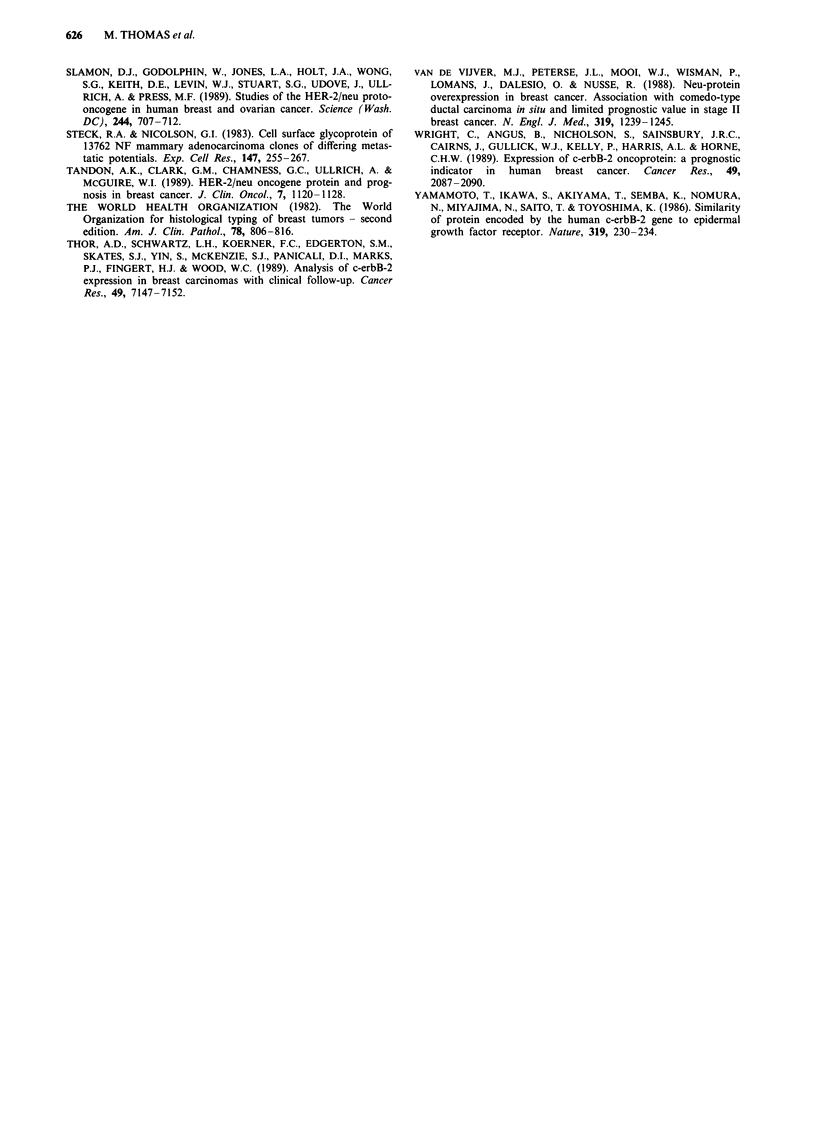


## References

[OCR_00625] Akiyama T., Sudo C., Ogawara H., Toyoshima K., Yamamoto T. (1986). The product of the human c-erbB-2 gene: a 185-kilodalton glycoprotein with tyrosine kinase activity.. Science.

[OCR_00633] Alam S. M., Whitford P., Cushley W., George W. D., Campbell A. M. (1990). Flow cytometric analysis of cell surface carbohydrates in metastatic human breast cancer.. Br J Cancer.

[OCR_00637] Altevogt P., Fogel M., Cheingsong-Popov R., Dennis J., Robinson P., Schirrmacher V. (1983). Different patterns of lectin binding and cell surface sialylation detected on related high- and low-metastatic tumor lines.. Cancer Res.

[OCR_00646] Berger M. S., Locher G. W., Saurer S., Gullick W. J., Waterfield M. D., Groner B., Hynes N. E. (1988). Correlation of c-erbB-2 gene amplification and protein expression in human breast carcinoma with nodal status and nuclear grading.. Cancer Res.

[OCR_00653] Bonadonna G., Valagussa P., Tancini G., Rossi A., Brambilla C., Zambetti M., Bignami P., Di Fronzo G., Silvestrini R. (1986). Current status of Milan adjuvant chemotherapy trials for node-positive and node-negative breast cancer.. NCI Monogr.

[OCR_00658] Borg A., Baldetorp B., Fernö M., Killander D., Olsson H., Sigurdsson H. (1991). ERBB2 amplification in breast cancer with a high rate of proliferation.. Oncogene.

[OCR_00663] Brooks S. A., Leathem A. J. (1991). Prediction of lymph node involvement in breast cancer by detection of altered glycosylation in the primary tumour.. Lancet.

[OCR_00668] Coussens L., Yang-Feng T. L., Liao Y. C., Chen E., Gray A., McGrath J., Seeburg P. H., Libermann T. A., Schlessinger J., Francke U. (1985). Tyrosine kinase receptor with extensive homology to EGF receptor shares chromosomal location with neu oncogene.. Science.

[OCR_00676] Fenlon S., Ellis I. O., Bell J., Todd J. H., Elston C. W., Blamey R. W. (1987). Helix pomatia and Ulex europeus lectin binding in human breast carcinoma.. J Pathol.

[OCR_00684] Fukutomi T., Itabashi M., Tsugane S., Yamamoto H., Nanasawa T., Hirota T. (1989). Prognostic contributions of Helix pomatia and carcinoembryonic antigen staining using histochemical techniques in breast carcinomas.. Jpn J Clin Oncol.

[OCR_00691] Furmanski P., Kirkland W. L., Gargala T., Rich M. A. (1981). Prognostic value of concanavalin A reactivity of primary human breast cancer cells.. Cancer Res.

[OCR_00707] Gullick W. J., Berger M. S., Bennett P. L., Rothbard J. B., Waterfield M. D. (1987). Expression of the c-erbB-2 protein in normal and transformed cells.. Int J Cancer.

[OCR_00712] Haybittle J. L., Blamey R. W., Elston C. W., Johnson J., Doyle P. J., Campbell F. C., Nicholson R. I., Griffiths K. (1982). A prognostic index in primary breast cancer.. Br J Cancer.

[OCR_00724] Klein P. J., Vierbuchen M., Wurz H., Schulz K. D., Newman R. A. (1981). Secretion-associated lectin-binding sites as a parameter of hormone dependence in mammary carcinoma.. Br J Cancer.

[OCR_00738] Leathem A. J., Brooks S. A. (1987). Predictive value of lectin binding on breast-cancer recurrence and survival.. Lancet.

[OCR_00743] Lovekin C., Ellis I. O., Locker A., Robertson J. F., Bell J., Nicholson R., Gullick W. J., Elston C. W., Blamey R. W. (1991). c-erbB-2 oncoprotein expression in primary and advanced breast cancer.. Br J Cancer.

[OCR_00751] McCann A. H., Dervan P. A., O'Regan M., Codd M. B., Gullick W. J., Tobin B. M., Carney D. N. (1991). Prognostic significance of c-erbB-2 and estrogen receptor status in human breast cancer.. Cancer Res.

[OCR_00755] Noguchi M., Koyasaki N., Ohta N., Kitagawa H., Earashi M., Thomas M., Miyazaki I., Mizukami Y. (1992). C-erbB-2 oncoprotein expression versus internal mammary lymph node metastases as additional prognostic factors in patients with axillary lymph node-positive breast cancer.. Cancer.

[OCR_00765] Paik S., Hazan R., Fisher E. R., Sass R. E., Fisher B., Redmond C., Schlessinger J., Lippman M. E., King C. R. (1990). Pathologic findings from the National Surgical Adjuvant Breast and Bowel Project: prognostic significance of erbB-2 protein overexpression in primary breast cancer.. J Clin Oncol.

[OCR_00770] Paterson M. C., Dietrich K. D., Danyluk J., Paterson A. H., Lees A. W., Jamil N., Hanson J., Jenkins H., Krause B. E., McBlain W. A. (1991). Correlation between c-erbB-2 amplification and risk of recurrent disease in node-negative breast cancer.. Cancer Res.

[OCR_00778] Semba K., Kamata N., Toyoshima K., Yamamoto T. (1985). A v-erbB-related protooncogene, c-erbB-2, is distinct from the c-erbB-1/epidermal growth factor-receptor gene and is amplified in a human salivary gland adenocarcinoma.. Proc Natl Acad Sci U S A.

[OCR_00787] Slamon D. J., Godolphin W., Jones L. A., Holt J. A., Wong S. G., Keith D. E., Levin W. J., Stuart S. G., Udove J., Ullrich A. (1989). Studies of the HER-2/neu proto-oncogene in human breast and ovarian cancer.. Science.

[OCR_00794] Steck P. A., Nicolson G. L. (1983). Cell surface glycoproteins of 13762NF mammary adenocarcinoma clones of differing metastatic potentials.. Exp Cell Res.

[OCR_00799] Tandon A. K., Clark G. M., Chamness G. C., Ullrich A., McGuire W. L. (1989). HER-2/neu oncogene protein and prognosis in breast cancer.. J Clin Oncol.

[OCR_00809] Thor A. D., Schwartz L. H., Koerner F. C., Edgerton S. M., Skates S. J., Yin S., McKenzie S. J., Panicali D. L., Marks P. J., Fingert H. J. (1989). Analysis of c-erbB-2 expression in breast carcinomas with clinical follow-up.. Cancer Res.

[OCR_00823] Wright C., Angus B., Nicholson S., Sainsbury J. R., Cairns J., Gullick W. J., Kelly P., Harris A. L., Horne C. H. (1989). Expression of c-erbB-2 oncoprotein: a prognostic indicator in human breast cancer.. Cancer Res.

[OCR_00830] Yamamoto T., Ikawa S., Akiyama T., Semba K., Nomura N., Miyajima N., Saito T., Toyoshima K. (1986). Similarity of protein encoded by the human c-erb-B-2 gene to epidermal growth factor receptor.. Nature.

[OCR_00816] van de Vijver M. J., Peterse J. L., Mooi W. J., Wisman P., Lomans J., Dalesio O., Nusse R. (1988). Neu-protein overexpression in breast cancer. Association with comedo-type ductal carcinoma in situ and limited prognostic value in stage II breast cancer.. N Engl J Med.

